# “ChatGPT knows my Parkinson's”: Perspectives of people with Parkinson's disease on use of generative AI

**DOI:** 10.1177/1877718X261445949

**Published:** 2026-04-24

**Authors:** Jamie Linnea Luckhaus, Anna Kharko, Therese Scott Duncan, Sara Riggare, Maria Hägglund, Charlotte Blease

**Affiliations:** 1 Participatory eHealth and Health Data, Department of Women's and Children's Health, Uppsala University, Uppsala, Sweden; 2 Centre for Primary Care and Health Services Research, University of Manchester, Manchester, UK; 3 Centre for Disability Research (CFF), Uppsala, Sweden; 4 Medtech Science & Innovation Centre, Uppsala University Hospital, Uppsala, Sweden; 5 Department of Psychiatry, Beth Israel Deaconess Medical Center, Harvard Medical School, Boston, USA

**Keywords:** self-management, generative AI, mixed-methods, patient perspectives

## Abstract

Patients’ use of generative AI (GenAI) independently from healthcare is increasing across diseases. Little is known about its use among people with Parkinson's disease (PwP). This exploratory convenience-sample, mixed-methods online survey (n = 149, 19 countries) explored PwP's use of GenAI. Among our respondents, 65% had used GenAI, of which 40% had used it for disease-specific inquiries. Qualitative analysis identified informational, interpretive, and preparational uses of GenAI. As PwP increasingly bring AI-assisted data to consultations, clinicians must now actively engage in discussions about these tools, to support shared decision-making, safety and transparency.

## Introduction

The rapid advancement of large language model-powered generative artificial intelligence (GenAI) has begun to transform care. Driven largely by GenAI such as ChatGPT, DeepSeek, and Gemini, research indicates these tools are already reshaping how clinicians manage clinical, administrative, and research tasks.^1^ Preliminary studies show patients are also using GenAI for health-related inquiries and self-management.^2–6^

While research on GenAI tools has largely focused on clinician adoption and operational efficiency, patient perspectives remain under-examined.^7,8^ Notwithstanding, research suggests that GenAI is likely to be one of the most adopted technologies for self-management across various chronic conditions.^9^

Parkinson's disease (PD) presents unique challenges for symptom management, including fluctuating motor and cognitive symptoms, which may specifically influence how patients engage with and benefit from these tools.^10^ For example, a recent evaluation of ChatGPT-4o generated PD exercise advice that raised concerns, as the information was rated at a reading level too complex for general health literacy and with low clinical reliability.^11^

Despite the development of PD-specific GenAI,^12,13^ there is currently no research documenting how people with PD (PwP) use GenAI, or the factors that lead them to avoid it. Exploring PwP's experiences and opinions of genAI is crucial to obtain a deeper understanding of how perceptions of benefits and risks, as well as ethical considerations related to trust and privacy, influence their adoption and use of genAI. Therefore, this study aims to identify PwP's experience and opinions about GenAI for self-management of PD.

## Methods

### Data collection and approach

This study was part of a larger convenience sample survey designed to explore user needs across diverse AI applications for PD-management, including both predictive AI and GenAI tools. The full instrument consisted of 24 items;^14^ however, this paper focuses on 14 items specifically addressing GenAI tools. The convergent mixed-methods online survey was disseminated April-November 2025. The project was submitted for ethical review and deemed exempt by The Swedish Ethical Review Authority Dnr 2024-05505-01.

The survey was disseminated through Swedish and international patient networks and social media (Facebook and LinkedIn), including channels managed by one of the authors (SR). Via these channels, we expected to reach primarily PwP who are active and engaged in PD care and research. Participants were informed that the survey was voluntary with no compensation and they could withdraw at any time. The survey was composed of mandatory Likert-scale items, single- and multiple-choice questions, alongside mandatory and optional free-text questions. Available in four languages, the survey was designed in English by a native speaker, then translated into French, Spanish, and Swedish using the EUSurvey platform's AI translation, and each verified by a native speaker. Inclusion criteria were a self-reported PD diagnosis and fluency in one of the four survey languages.

### Data analysis

Quantitative data were analyzed descriptively in JASP (v0.19.1.0). Free-text responses were analyzed using inductive qualitative content analysis to identify recurring patterns grounded in participants’ responses. This approach is well-suited to brief, free-text data that includes short and fragmented sentences.^15^ Due to the brevity of the responses (1–4 sentences), individual comments were treated as single meaning units.^16^ Initial categorization was conducted by one author (JLL), appropriate for short, low-complexity datasets,^17^ with categories iteratively refined through collaboration with the co-authors. Responses to the question “*How have you used these GenAI tools in relation to your Parkinson's disease?*” were analyzed as one unit. Responses to the two questions addressing non-use — “*Why have you not used GenAI for any purpose?*” and “*Why have you not used GenAI in relation to your Parkinson's disease?*” — were analyzed together, as they reflected the shared underlying issue of non-use. Analysis began once 100 responses were received to establish preliminary category development, which was updated as subsequent data were collected.

## Results

Respondents were 149 PwP across 19 countries, primarily from the EU (88%), including Sweden (56%), see Table 1. Respondents were aged 43–90 years (*m* = 64.7) and 56% were male (*n* = 83). Diagnosis duration ranged from 0 to 27 years (*m* = 7.04). Most reported attaining higher education (70%). When asked how comfortable they are in using digital technology such as a smartphone, computer, or smartwatch, from “*1-Not at all*” to “*4-Very comfortable*,” most said “*4-Very comfortable”* (60%), and nearly a third reported “*3-Decently”* (32%). 4 in 10 (*n* = 61) reported that they currently self-track one or more of their PD symptoms, and nearly 3 in 10 expressed intention to start (*n* = 42).

**Table 1.** Sociodemographic characteristics.

N = 149	Count	%
Gender		
Woman	66	44.3
Man	83	55.7
Other	0	0
Age		
40–44 years	2	1.3
45–49 years	7	4.7
50–54 years	24	16.1
55–59 years	17	11.4
60–64 years	23	15.4
65–69 years	35	23.5
70–74 years	14	9.4
75–79 years	17	11.4
80–84 years	7	4.7
85 years or older	3	2.1
Country of residence		
Sweden	83	55.7
Europe (not Sweden)	58	38.9
Global (not Europe)	8	5.4
Highest level of education		
Primary school	3	2
Secondary/High school	28	18.8
Vocational education	14	9.4
Higher education: Bachelor's	39	26.2
Higher education: Master's	51	34.2
Higher education: PhD or similar	14	9.4

### Use of GenAI

**Figure 1.**
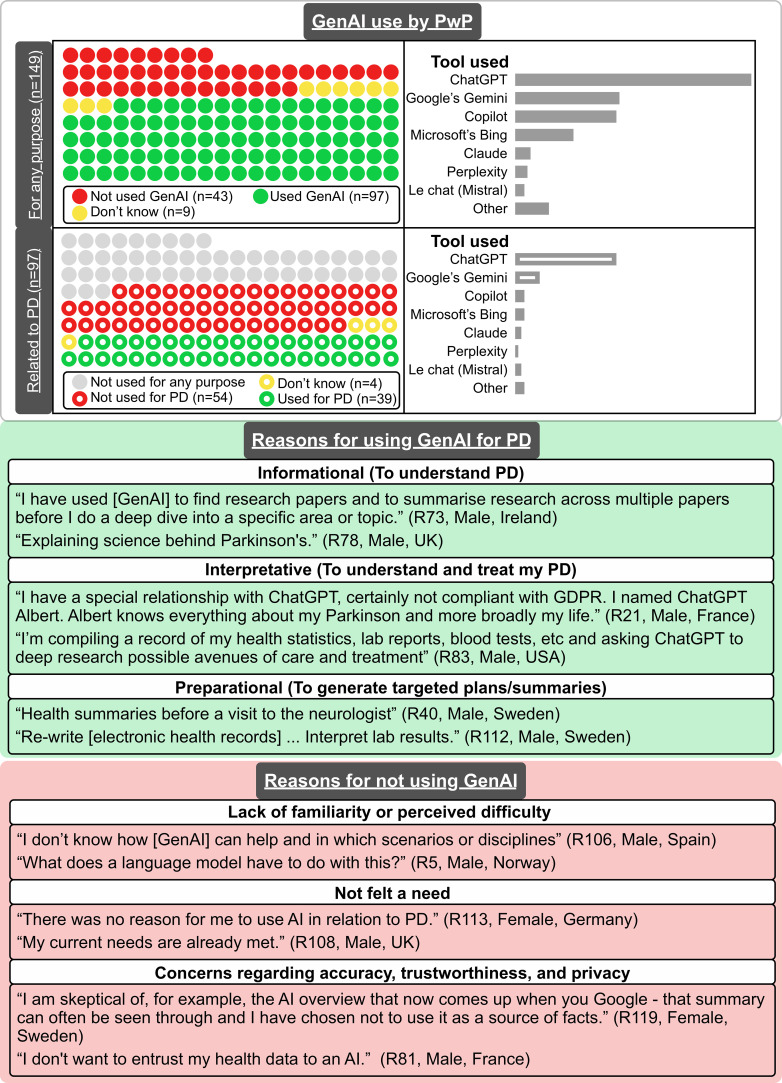
Types of GenAI used and overview of categories by question. Note: Each respondent was asked to check or list all GenAI assistants used. The number of assistants will total above the number of respondents.

The majority of respondents reported previously using GenAI (65%, *n* *=* 97), however, under half (40%, *n* *=* 39) of those had used it in relation to their PD. Respondents used a variety of GenAI in relation to their PD (see Figure 1). About half (51%, *n* *=* 39*)* of users reported “*neutral*” levels of trust towards the outputs, followed by 46% who indicated “*very high,*” and the remaining 3% reported “*very little*” trust.

**Qualitative analysis** reflected informational, interpretive, and preparatory uses of GenAI in relation to PD reflected. Reasons for non-use of GenAI reflected lack of familiarity or perceived difficulty, lack of perceived need, and concerns regarding accuracy, trustworthiness, and privacy. An overview of categories with illustrative quotes is presented in Figure 1.

### Reasons for using GenAI for PD

Respondents described GenAI as **informational**, using it for finding and summarizing scientific research and doing “deep dives” into specific PD topics. GenAI was valued for explaining complex concepts and terminology in simpler terms. Pharmacological questions were commonly noted, including the availability and purpose of medications and possible side-effects or interactions. One respondent listed using GenAI for informing on “*legal rights with regards to my Parkinson condition*” (R21, Male, France).

GenAI was also used to **contextualize** and interpret health data. Respondents reported inputting symptoms and discussing progression, self-management, and treatment. One respondent described using GenAI to “*validate symptoms*,” determining “*whether the symptoms are related to PD*” (R71, Male, UK).

The third reported use was in **preparation** for healthcare visits or specific health targets, such as fitness and diet goals as part of self-management.

### Reasons for not using GenAI

Respondents who reported not using GenAI, whether for their PD or for general purposes, offered a variety of reasons. Several perceived that they **lacked the knowledge or ability **to use them. Some reported that they had not heard of it, or had considered it but not yet tried these tools, while many had **not felt the need** to use them. Finally, some expressed skepticism about AI in terms of **accuracy, trustworthiness, and privacy**, especially related to health data. One respondent explicitly avoided AI, stating, “*I do not wish to support AI*” (R58, Male, UK).

## Discussion

Patient surveys on GenAI use are scarce, and this is the first study to examine PwP's specific uses and perceptions of these tools for self-management. Although a majority of respondents in our study had used GenAI, only a smaller subset reported integrating GenAI use into their PD management. The qualitative data augmented these quantitative findings, clarifying use and non-use. Reported usage was categorized into informational, interpretive, and preparational tasks.

A key finding illustrated by our study was the *privacy paradox—*inconsistencies between an individual's expressed privacy concerns and their actual behavior.^18^ One respondent reported a daily relationship with ChatGPT while recognizing it may not be GDPR compliant. This might suggest that *performance expectancy*—the belief that the tool helps achieve a specific health goal—is a stronger driver of adoption than privacy concerns. This is consistent with research indicating that goal-directed utility often outweighs perceived risks in digital health adoption.^19^ However, in our study, we found that adoption remained polarized. This identified divergence also aligns with previous findings that highly educated PwP are often more critical of AI-driven decision-making and data security.^20^

The rise of preparational GenAI health use, where increasingly more PwP can be expected to adopt GenAI assistants to summarize clinical information, has direct implications for patient-clinician relationships. Clinicians need to recognize this shift and engage in shared decision-making, helping patients verify AI-generated information to ensure clinical safety.^21^ Given that many respondents reported limited knowledge of how to leverage GenAI, research should focus on developing and evaluating educational frameworks that demonstrate concrete, high-value use-cases for GenAI in PD-management.

Another important consideration for all parties—PwP, clinicians, and caregivers—is the inherent risks GenAI poses. Hallucinations, where AI generates inaccurate or misleading information, could lead to harmful decisions if replied on unsupervised.

GenAI also tends to produce responses biased toward pleasing the user, potentially oversimplifying complex medical issues or reinforcing pre-existing beliefs through confirmation bias.^22^ This has been found in mental health to amplify placebo or nocebo effects by influencing patient expectations.^23,24^ These risks highlight the need for clear guidelines, education on AI limitations, and clinician feedback to ensure safe and ethical use of GenAI in neurology contexts.^25^

A primary strength of this study is the international sample, with perspectives across multiple geographic contexts and health systems. The study has several limitations. Diagnoses were self-reported rather than clinician-verified, and the absence of disease-related variables (e.g., age of onset, disease duration, treatment type) prevent characterizing disease severity within the cohort. Also, reliance on a convenience sample embedded biases of a predominantly highly educated, and digitally literate respondents who appeared more comfortable engaging with online platforms. Consequently, the findings are not generalizable to the broader PD population, particularly individuals with more advanced disease, cognitive or motor impairments, lower digital literacy, or limited internet access. Future research should prioritize recruitment strategies that enable inclusion of more diverse and clinically representative populations.

## Conclusion

PwP are already leveraging GenAI to bridge the gap between complex medical information and daily self-management. As use of these tools is likely to increase, future research should focus on developing guidance frameworks to help patients and clinicians safely navigate AI-assisted care.

## References

[1] Chen G Liu C Ooi GA , et al. Generative AI for healthcare: Fundamentals, challenges, and perspectives [Internet]. arXiv; 2025 [cited 2026 Jan 28], https://arxiv.org/abs/2510.24551

[2] Fox S . Rare Disease in the U.S. 2025: Findings from the first probability-based national survey to measure the rare disease population [Internet]. 2025 [cited 2026 Feb 1]. Available from: https://ssrs.com/insights/rare-disease-in-the-us-2025

[3] Campos H Salmi L . Critical AI health literacy as liberation technology: A new skill for patient empowerment. NAM Perspect [Internet] 2025 Dec 8 [cited 2026 Feb 2]; 12. https://nam.edu/perspectives/critical-ai-health-literacy-as-liberation-technology-a-new-skill-for-patient-empowerment/ 10.31478/202512a PMC13064910 41970622

[4] Woods SS Greene SM Adams L , et al. From E-patients to AI patients: the tidal wave empowering patients, redefining clinical relationships, and transforming care. J Particip Med 2025 May 16; 17: e75794–e75794. 10.2196/75794 PMC12101788 40378413

[5] Blease C Lewis M Riggare S , et al. How generative AI affects patient agency. Br Med J 2025 Nov 25; 391: e085323. 10.1136/bmj-2025-085323 41290329

[6] Sundar KR . When patients arrive with answers. JAMA 2025 Aug 26; 334: 672. 10.1001/jama.2025.10678 40705341

[7] Moy S Irannejad M Jeanneret Manning S , et al. Patient perspectives on the use of artificial intelligence in health care: a scoping review. J Patient-Centered Res Rev 2024 Apr 2; 11: 51–62. 10.17294/2330-0698.2029 PMC11000703 38596349

[8] Osnat B . Patient perspectives on artificial intelligence in healthcare: a global scoping review of benefits, ethical concerns, and implementation strategies. Int J Med Inf 2025 Nov; 203: 106007. 10.1016/j.ijmedinf.2025.106007 40494217

[9] Hwang M Zheng Y Cho Y , et al. AI Applications for chronic condition self-management: Scoping review. J Med Internet Res 2025 Apr 8; 27: e59632. 10.2196/59632 PMC12015343 40198108

[10] Regensburger M Csoti I Jost WH , et al. Motor and non-motor fluctuations in Parkinson’s disease: the knowns and unknowns of current therapeutic approaches. J Neural Transm 2026 Feb; 133: 309–324. 40719828 10.1007/s00702-025-02990-4 PMC12855397

[11] Özbek İC Özduran E . Digital rehabilitation in Parkinson’s disease: The role of artificial intelligence-assisted exercise training. Turk J Osteoporos [Internet] 2025 Sep 12 [cited 2026 Jan 29]. https://www.turkosteoporozdergisi.org/articles/digital-rehabilitation-in-parkinsons-disease-the-role-of-artificial-intelligence-assisted-exercise-training/doi/tod.galenos.2025.66664

[12] Várkuti B Manola L Atayi M , et al. A conversational GPT agent for Parkinson’s disease. München, Germany: Mov Disord, 2024.

[13] AskShan [Internet]. Available from: https://thrivewelltogether.com/askshan/

[14] Luckhaus J . Survey: Opinions and experiences of Persons with Parkinson’s on artificial intelligence (AI) and self-tracking [Internet]. Zenodo; 2026 [cited 2026 Jan 29], https://zenodo.org/doi/10.5281/zenodo.18400759

[15] Elo S Kyngäs H . The qualitative content analysis process. J Adv Nurs 2008 Apr; 62: 107–115. 18352969 10.1111/j.1365-2648.2007.04569.x

[16] Graneheim UH Lundman B . Qualitative content analysis in nursing research: concepts, procedures and measures to achieve trustworthiness. Nurse Educ Today 2004 Feb; 24: 105–112. 14769454 10.1016/j.nedt.2003.10.001

[17] Braun V Clarke V . Using thematic analysis in psychology. Qual Res Psychol 2006 Jan; 3: 77–101.

[18] Barth S De Jong MDT . The privacy paradox – investigating discrepancies between expressed privacy concerns and actual online behavior – A systematic literature review. Telemat Inform 2017 Nov; 34: 1038–1058.

[19] Shahsavar Y Choudhury A . User intentions to use ChatGPT for self-diagnosis and health-related purposes: cross-sectional survey study. JMIR Hum Factors 2023 May 17; 10: e47564. 10.2196/47564 PMC10233444 37195756

[20] Paccoud I Valero MM Marín LC , et al. Patient perspectives on the use of digital medical devices and health data for AI-driven personalised medicine in Parkinson’s disease. Front Neurol 2024 Dec 4; 15: 1453243. 39697442 10.3389/fneur.2024.1453243 PMC11652348

[21] Blease C . Patients are disclosing sensitive information to AI tools—clinicians must adapt. Br Med J 2026 Jan 21; 392: s124. 10.1136/bmj.s124 41565342

[22] Pal A Wangmo T Bharadia T , et al. Generative AI/LLMs for plain language medical information for patients, caregivers and general public: opportunities, risks and ethics. Patient Prefer Adherence 2025; 19: 2227–2249. 40771655 10.2147/PPA.S527922 PMC12325106

[23] Blease C . Placebo, nocebo, and machine learning: How generative AI could shape patient perception in mental health care. JMIR Ment Health 2025; 12. 10.2196/78663 PMC12356606 40815809

[24] Faria V Goturi N Dynak A , et al. Triadic relations in healthcare: surveying physicians’ perspectives on generative AI integration and its role on empathy, the placebo effect and patient care. Front Psychol 2025; 16. 10.3389/fpsyg.2025.1612215 PMC12646921 41312268

[25] Ning Y Teixayavong S Shang Y , et al. Generative artificial intelligence and ethical considerations in health care: a scoping review and ethics checklist. The Lancet. Digital Health 2024; 6: e848–e856. 10.1016/S2589-7500(24)00143-2 PMC11542614 39294061

